# Serologic Evidence of Hemorrhagic Fever Virus Spillover in Rural Liberia

**DOI:** 10.1093/ofid/ofag100

**Published:** 2026-03-04

**Authors:** Adam M Schafer, Emmanuel Kerkula, Chanhwa Lee, Alfred Flomo, Amara Fofana, Stanley Kerkula, Thomas Sumo, Alexander Sampson, Samuel Vouh, Fred Flomo, McKenzie A Colt, Katie R Mollan, Taylor J Krajewski, Eleanor Rose Watts, Catherine Nimely, Randal J Schoepp, Keersten Ricks, Minnie Ricks, Jefferson Sibley, Jacob A Dillard, David A Wohl, William A Fischer

**Affiliations:** Institute for Global Health and Infectious Diseases, The University of North Carolina, Chapel Hill, North Carolina, USA; Institute for Global Health and Infectious Diseases, Project Liberia, The University of North Carolina, Bong, Liberia; Department of Biostatistics, Gillings School of Global Public Health, The University of North Carolina, Chapel Hill, North Carolina, USA; Institute for Global Health and Infectious Diseases, Project Liberia, The University of North Carolina, Bong, Liberia; Institute for Global Health and Infectious Diseases, Project Liberia, The University of North Carolina, Bong, Liberia; Institute for Global Health and Infectious Diseases, Project Liberia, The University of North Carolina, Bong, Liberia; Institute for Global Health and Infectious Diseases, Project Liberia, The University of North Carolina, Bong, Liberia; Institute for Global Health and Infectious Diseases, Project Liberia, The University of North Carolina, Bong, Liberia; Institute for Global Health and Infectious Diseases, Project Liberia, The University of North Carolina, Bong, Liberia; Institute for Global Health and Infectious Diseases, Project Liberia, The University of North Carolina, Bong, Liberia; Institute for Global Health and Infectious Diseases, Project Liberia, The University of North Carolina, Bong, Liberia; Department of Biostatistics, Gillings School of Global Public Health, The University of North Carolina, Chapel Hill, North Carolina, USA; Department of Biostatistics, Gillings School of Global Public Health, The University of North Carolina, Chapel Hill, North Carolina, USA; Vaccine Development and Evaluation Centre, UK Health Security Agency, Andover, England, UK; Institute for Global Health and Infectious Diseases, Project Liberia, The University of North Carolina, Bong, Liberia; Diagnostic Systems Division, US Army Medical Research Institute of Infectious Diseases, Fort Detrick, Maryland, USA; Diagnostic Systems Division, US Army Medical Research Institute of Infectious Diseases, Fort Detrick, Maryland, USA; Department of Pediatrics, Phebe Hospital, Bong, Liberia; Department of Pediatrics, Phebe Hospital, Bong, Liberia; Department of Surgery, Phebe Hospital, Bong, Liberia; Department of Microbiology and Immunology, The University of North Carolina School of Medicine, Chapel Hill, North Carolina, USA; Institute for Global Health and Infectious Diseases, The University of North Carolina, Chapel Hill, North Carolina, USA; Institute for Global Health and Infectious Diseases, The University of North Carolina, Chapel Hill, North Carolina, USA

**Keywords:** ebolavirus, flavivirus, Lassa virus, Liberia, Marburg virus

## Abstract

**Background:**

Outbreaks of zoonotic emerging infectious diseases, including viral hemorrhagic fevers, are increasing in frequency. Clinical detection remains challenging due to the lack of pathognomonic signs or symptoms and limited access to diagnostics. To better understand the prevalence of prior exposure to viral hemorrhagic fever viruses, serum from community participants in rural Liberia was tested for immunoglobulin G antibodies.

**Methods:**

Serum collected from individuals enrolled in the ENABLE study, an observational study of Lassa fever virus incidence and seroprevalence, were analyzed for immunoglobulin G against Ebola virus, Marburg virus, Lassa virus, Rift Valley Fever virus, Crimean-Congo hemorrhagic fever virus, pan-alphavirus, and pan-flavivirus by MAGPIX, a multiplex immune assay. Associations with seropositivity were evaluated using questionnaires that included demographic, animal, and environmental exposure information.

**Results:**

Eighty-eight percent of samples from 456 participants tested positive for ≥1 of the viral antibodies with a majority (63%) having antibodies to ≥2 viruses. Seropositivity was highest for Lassa virus (67%) followed by pan-flavivirus (51%), pan-alphavirus (35%), Crimean-Congo hemorrhagic fever virus (24%), Ebolavirus (13%), Rift Valley Fever virus (9%), and Marburg virus (8%). Older age, sex (variable by pathogen), and exposure to cats and rats were associated with seropositivity.

**Conclusions:**

These findings demonstrate a significant spillover of filoviruses, bunyaviruses, flaviviruses, and alphaviruses in rural Liberia in contrast with an absence of detected outbreaks. These data support the need for enhanced surveillance and understanding of the ecological and behavioral risk factors for zoonotic spillover events, across a spectrum of disease presentation, given their potential and ongoing threat to global public health.

## Teaser

Sero-surveillance of a rural Liberian population revealed higher than expected exposure to viral hemorrhagic fever viruses, with a lack of reported outbreaks, highlighting the need for improved surveillance and diagnostics.

## BACKGROUND

Approximately 60% of human infectious diseases originate in animals and an estimated 75% of emerging pathogens are zoonotic [1–3]. Population growth, human encroachment on wild habitats, changes in land use, increased animal production for food, and climate change have intensified interactions between humans and vectors for pathogen transmission [2]. Although zoonotic outbreaks occur globally, in Africa, outbreaks of disease caused by pathogens transmitted from animals to humans are becoming increasingly frequent. From 2012 to 2022, zoonotic outbreaks in Africa increased by 63% compared to the previous decade, with 70% caused by pathogens associated with viral hemorrhagic fevers (VHFs) [4]. West Africa with its rapidly expanding population, urbanization, and deforestation has experienced spillovers of several high consequence human pathogens including the VHFs Ebola virus (EBOV), and Lassa virus (LASV), which is endemic to this region.

VHFs are a group of severe viral infections primarily caused by 4 families of viruses: *Arenaviridae*, *Bunyaviridae*, *Filoviridae,* and *Flaviviridae* including EBOV, LASV, Marburg virus (MARV), Rift Valley Fever virus (RVFV), Yellow Fever virus (YFV), and Crimean-Congo hemorrhagic fever virus (CCHFV) [5]. Case fatality rates have historically reached as high as 65% for Lassa fever and 90% for Ebola virus disease [6–9]. High case fatality rates, limited or absent vaccines/therapeutics, and scarce global diagnostic infrastructure, combined with the potential for rapid spread far from the outbreak origin, make this group of pathogens a threat to global public health.

However, clinical detection of VHFs is challenging on multiple levels in resource-limited settings like those in West Africa, where healthcare services can be sparse and diagnostics are not routinely available. Furthermore, the lack of pathognomonic symptoms and an increasingly recognized broad spectrum of illness severity lead to under detection of these pathogens or attribution to more prevalent infectious diseases, particularly malaria. As a result, the index cases of outbreaks caused by these pathogens are typically missed. Failure to detect cases of VHFs and other zoonoses also perpetuates a vicious cycle in which the underappreciation of their presence in communities and regions results in appropriate diagnostics not being deployed. To gain a better understanding of spillover risk in West Africa, we examined serological evidence of prior exposure to VHFs and other major emerging zoonotic pathogens in people living in a rural and forested region of Liberia who were participating in a 24-month observational study of Lassa fever sero-incidence and sero-prevalence.

## METHODS

### Study Participants and Setting

Serum samples were collected from Liberian participants enrolled in the Coalition for Epidemic Preparedness Innovations ENABLE study at the time of study entry, which was conducted in 3 rural communities in the rural center of the country [10]. These community sites were selected due to historically high rates of Lassa fever. Eligible participants were ≥2 years of age, residents in the community for at least 6 months before study enrollment and for the duration of the 24-month study, and reported no fever at the time of enrollment. For this analysis, participants from two of the studied non-contiguous communities, Phebe Airstrip and Suakoko, were purposefully selected to achieve balanced sex distribution and include participants across the age lifespan.

### Patient Consent Statement

The study was approved by the UNC School of Medicine Institutional Review Board and The University of Liberia Institutional Review. Written informed consent was obtained from all participants.

### Serological Testing

Blood was collected from community sites using serum separation tubes (5 mL per participant), stored at 2–8 °C and transported within 4 hours to the laboratory for processing and long-term storage at −80 °C. Serological testing was performed using the MAGPIX assay for the following antigens (virus like particles [VLP] and recombinant proteins): Pan-Flavivirus VLP with Dengue virus glycoprotein (Native Antigen Company), EBOV nucleoprotein (University of Hawaii), Pan-alpha VLP with Chikungunya virus glycoprotein (Native Antigen Company), CCHFV nucleoprotein (USAMRIID), LASV nucleoprotein (University of Hawaii), MARV glycoprotein (Native Antigen Company), and RVFV nucleoprotein (Native Antigen Company) [11]. The pan-Flavi VLP used can detect immunoglobulin G (IgG) antibodies to DENV, West Nile virus, YFV, Japanese encephalitis virus, and tickborne encephalitis virus [11]. The pan-alpha VLP can detect IgG antibodies to Sindbis virus, CHIKV, O’nyong-nyong virus, Venezuelan equine encephalitis virus, Western equine encephalitis virus, and Eastern equine encephalitis virus [11]. Study samples were run alongside a positive control consisting of a pool of known IgG-positive human convalescent serum and a negative control consisting of known negative American human serum. See supplemental text for more detailed MAGPIX methodology.

The results of the assay were reported in units of mean fluorescent intensity. Signal-to-noise ratios (SNR) were calculated by normalizing the raw signal of each sample against the negative control. The sample was positive if its SNR value was ≥20. This cutoff provides a more robust alternative to using a fourfold increase over baseline, a method that is difficult to apply to populations with unknown seroprevalence [11–13]. SNR of 20 closely mimics a formal limit of quantitation (α_negative + 10*SD_negative) and standardizes the interpretation of positivity for each of the viral assays. Previous MAGPIX studies have also used an SNR threshold of 20 [12, 14, 15]. We additionally analyzed LASV IgG serology using a SNR ≥40, which has been associated with neutralizing LASV antibodies (K. Ricks, personal communication, 29 April 2025). No SNR thresholds for neutralizing antibodies have been established for the other targeted viruses.

### Questionnaires

Demographic and potential risk factors for LASV infection that included animal exposures were collected at ENABLE study enrollment by research assistants using tablet computers and included age, sex, number of persons living in the household, food storage methods, presence of rat holes around the house, house construction materials, trash disposal methods, and animals kept around the house. Household-level features were reported by the household's head or one main informant.

### Statistical Analysis

Unadjusted seroprevalence for each virus was estimated along with a 95% confidence interval (CI) using a logistic intercept-only generalized estimating equation (GEE) with an exchangeable correlation structure to account for multiple participants per household. Associations between covariates measured at enrollment and virus seropositivity were assessed separately for each virus using bivariate logistic GEE models with an exchangeable correlation structure. Bivariate analyses with at least 10 households in both the index and reference groups accounted for within-household correlation and a small cluster number correction was applied. Because GEE can underestimate the variance when there are few clusters, bivariate analyses with fewer than 10 households in either group did not account for within-household correlation [16]. Seropositivity was modeled as the outcome and each covariate as the predictor. We estimated a prevalence difference (PD) and corresponding 95% CI by applying the Delta method. Correlations in seropositivity across the seven viruses were calculated using the Phi coefficient. Missing data were rare and handled via complete case analysis. All analyses were conducted using R version 4.2.3.

## RESULTS

### Participant Demographics

Between April 2021 and December 2023, 5005 participants were enrolled in the ENABLE study. Baseline serum samples from 456 participants (from 96 households) were tested. Median age was 18 years (interquartile range: 11–31) and 45% were male (Table 1). Most participants lived in households with 8 or more individuals. Four participants reported prior Lassa fever and 3 participants reported having EBOV disease before enrollment.

**Table 1.** Demographics of Participants From Phebe Airstrip and Suakoko

	PHE	SUA	Overall
Characteristic	n & 411	n = 45	n = 456
Age, n (%)	…	…	…
2–5	42 (10%)	3 (6.7%)	45 (9.9%)
6–11	65 (16%)	5 (11%)	70 (15%)
12–19	121 (30%)	15 (33%)	136 (30%)
20–29	74 (18%)	6 (13%)	80 (18%)
30–39	43 (10%)	2 (4.4%)	45 (9.9%)
40–49	25 (6.1%)	0 (0%)	25 (5.5%)
50–59	14 (3.4%)	9 (20%)	23 (5.1%)
60+	26 (6.3%)	5 (11%)	31 (6.8%)
Missing	1	…	1
Sex, n (%)	…	…	…
Female	224 (55%)	29 (64%)	253 (55%)
Male	187 (45%)	16 (36%)	203 (45%)
Number of persons living in the household, n (%)	…	…	…
1–7	112 (27%)	5 (11%)	117 (26%)
8–10	131 (32%)	18 (40%)	149 (33%)
11–14	84 (20%)	1 (2.2%)	85 (19%)
15+	84 (20%)	21 (47%)	105 (23%)
Is food stored/dried out in the open?, n = yes (%)	189 (46%)	31 (69%)	220 (48%)
Are there rat holes around or close to the house?, n = yes (%)	380 (92%)	24 (53%)	404 (89%)
What are the house walls made of?, n = yes (%)	…	…	…
Cement/brick	264 (64%)	30 (67%)	294 (64%)
Mud/other	147 (36%)	15 (33%)	162 (36%)
Trash storage methods, n = yes (%)	…	…	…
Stored in open bins inside	23 (5.6%)	1 (2.2%)	24 (5.3%)
Stored in open bins outside	238 (57.9%)	15 (33.3%)	253 (55.5%)
Trash pile close by the house	33 (8.0%)	0 (0.0%)	33 (7.2%)
Trash pile not close to the house	106 (25.8%)	21 (46.7%)	127 (27.9%)
Animal exposures, n = yes (%)	…	…	…
Dog	142 (34.5%)	30 (66.7%)	172 (37.7%)
Cat	84 (20.4%)	0 (0.0%)	84 (18.4%)
Chicken	289 (70.3%)	40 (88.9%)	329 (72.1%)
Other^a^	55 (13.4%)	0 (0.0%)	55 (12.1%)
None	44 (10.7%)	10 (22.2%)	54 (11.8%)

Abbreviations: PHE, Phebe Airstrip; SUA, Suakoko.

^a^Other animals were unspecified with the exception of 4 individuals who specified ducks.

### Prevalence of Anti-viral IgG Antibodies

At an SNR of ≥20, the prevalence of seropositivity was greatest for LASV (67%; CI, 62–71%) followed by pan-flavivirus (51%; CI, 46–57%), pan-alphavirus (35%; CI, 31–41%), CCHFV (24%; CI, 19–29%), EBOV (13%; CI, 10–17%), RVFV (9%; CI, 7–13%), and MARV (8%; CI, 6–11%) (Table 2). Analyzing the LASV data at the neutralizing antibody threshold of SNR ≥40, LASV seropositivity was 56% (CI, 51–61%) (Table 2). See Supplementary figure 1 for distribution plot of MFI-SNR values.

**Table 2.** Seropositivity Rates of Each Target Virus Among Participant Samples Expressed as Percentage With CI; Rates Were Reported for Phoebe Airstrip, Suakoko, and Overall

	n & number of seropositive (95% CI)		
Virus	PHE (n = 411)	SUA (n = 45)	Overall (n = 456)
LASV^a^	n = 280, 68% (62%–73%)	n = 25, 56% (42%–75%)	n = 305, 67% (62%–71%)
LASV^b^	n = 245, 60% (54%–65%)	n = 11, 24% (13%–41%)	n = 256, 56% (51%–61%)
Panflavi	n = 224, 55% (48%–60%)	n = 10, 22% (13%–39%)	n = 234, 51% (46%–57%)
Panalpha	n = 140, 34% (30%–40%)	n = 20, 44% (32%–70%)	n = 160, 35% (31%–41%)
CCHFV	n = 99, 24% (19%–30%)	n = 9, 20% (11%–32%)	n = 108, 24% (19%–29%)
EBOV	n = 58, 14% (11%–18%)	n = 1, 2% (0%–16%)	n = 59, 13% (10%–17%)
RVFV	n = 38, 9% (7%–13%)	n = 5, 11% (5%–21%)	n = 43, 9% (7%–13%)
MARV	n = 34, 8% (5%–12%)	n = 3, 7% (3%–17%)	n = 37, 8% (6%–11%)

Abbreviations: CCHFV, Crimean-Congo hemorrhagic fever virus; EBOV, Ebola virus; LASV, Lassa virus; MARV, Marburg virus; PHE, Phebe Airstrip; RVFV, Rift Valley Fever virus; SUA, Suakoko.

^a^LASV seropositivity using signal-to-noise ratio of 20.

^b^LASV seropositivity using signal-to-noise of 40, which is associated with neutralizing antibodies.

### Factors Associated With Seropositivity

Seropositivity rates for LASV and alphaviruses both correlated with older age. LASV seropositivity was 52.9% in children 6–11 years of age and peaked at 88% in adults aged 40–49 (Supplementary Figure 2.1). Similarly, alphavirus seropositivity peaked at 88% in participants aged 40–49 (Supplementary Figure 2.2). Age-related trends were not observed for the lower prevalence pathogens EBOV, MARV, CCHFV, RVFV, or pan-flavivirus.

Biological sex-related differences in seroprevalence were noted for RVFV and flaviviruses. For RVFV, seropositivity was more prevalent in males than females (PD: 7.9%; CI, 2.2%–13.5%) (Supplementary Figures 2.3). For flaviviruses, seropositivity was more prevalent among females compared to males (PD: −10.5%; CI, −19.7 to −1.4%) (Supplementary Figure 2.4). No significant sex differences were found for LASV, MARV, EBOV, CCHFV, or pan-alphaviruses seropositivity.

Correlations with seropositivity were noted with exposure to cats and rats but no other animals surveyed. CCHFV (PD: 13.2%; CI, 1.1%–25.4%) and MARV (PD: 9.4%; CI, .3%–18.4%) seropositivity was more prevalent among individuals with cat exposures than those who reported no cat exposures (Supplementary Figure 2.5 and 2.6). Conversely, alphavirus seropositivity was less in those with cat exposures (PD: −19.5%; CI, −31.4 to −7.6%) (Supplementary Figure 2.2). MARV seropositivity was less prevalent in those reporting exposures to animals listed as other which, except for 4 individuals, were not specified (all 4 reported duck exposures) (Supplementary Figure 2.6). Households reporting ratholes trended toward higher seropositivity for LASV (14.5%; CI, −2.2% to 31.1%) (Supplementary Figure 2.1). RVFV seropositivity prevalence was less in individuals reporting no animal exposures (PD: −6.7%; CI, −12.8 to −.6%) (Supplementary Figure 2.3). No notable correlations were observed for EBOV and the factors studied here (Supplementary Figure 2.7).

### Evidence of Exposure to Multiple Viral Pathogens

**Figure 1.**
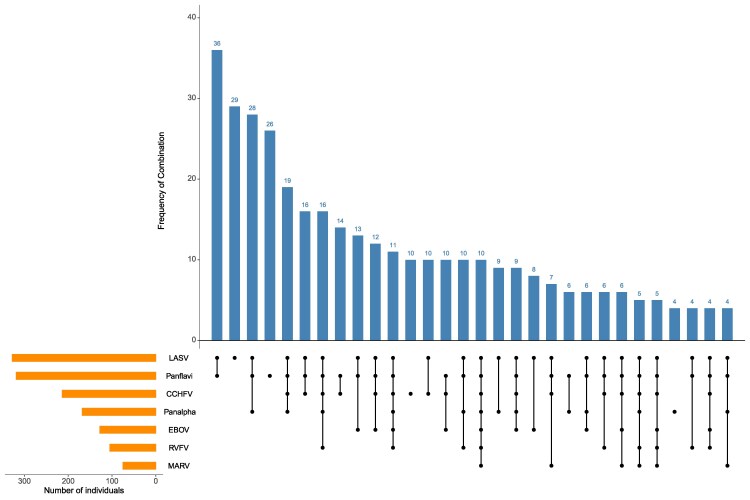
Seropositivity combinations plot. Combinations of virus seropositivity displayed along the x-axis (black connected lines) and number of serum samples in each combination along the y-axis (blue bars). Set size (number of seropositive individuals displayed on figure) for each virus are shown (orange bars). Virus combinations including fewer than n & 3 serum samples were omitted for brevity. Lassa virus (LASV), pan-flavivirus, Crimean-Congo hemorrhagic fever virus (CCHFV), pan-alphavirus, Ebola virus (EBOV), Rift Valley fever virus (RVFV) and Marburg virus (MARV).

**Figure 2.**
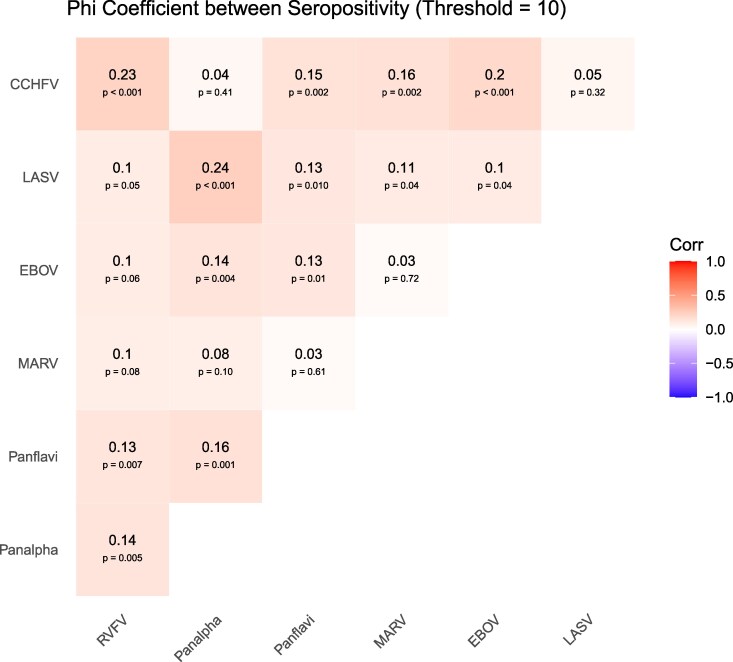
Bivariate Phi coefficients between virus seropositivity for dichotomized values. Values range from −1 (negative correlation), 0 (no correlation) and 1 (positive correlation). A *P* value is displayed under each Phi coefficient.

Using an SNR of ≥20, 88% of samples were seropositive for at least 1 of the antibodies tested (Table 3). Participants were seropositive to a mean of 2.1 tested pathogens with 64% positive for 2 or more (Table 3). Only 54 (12%) were seronegative for all 7 viruses tested (Table 3). Patterns of the most common co-seropositivity included LASV along with flavivirus followed by the combination of LASV, flaviviruses, and alphaviruses (Figure 1). Analysis of Phi estimates showed the strongest correlation between LASV and alphaviruses (0.24, *P* < .001), CCHFV and RVFV (0.23, *P* < .001), and CCHFV and EBOV (0.2, *P* < .001) (Figure 2). A sensitivity analysis using absolute antibody concentrations found stronger correlations (Supplementary Figure 3).

**Table 3.** Distribution of Seropositive Tests per Sample, 7 Tests Were Included (LASV, RVFV, CCHFV, EBOV, MARV, Pan-alphavirus, and Pan-flavivirus). Rates Were Reported for Phoebe Airstrip, Suakoko, and Overall

Region	Total Samples	Number of Seropositive Tests Per Sample								Mean Number of Seropositive Tests	Median Number of Seropositive Tests
		0	1	2	3	4	5	6	7		
Overall	456	54 (12%)	114 (25%)	136 (30%)	85 (19%)	41 (9%)	17 (4%)	7 (2%)	2 (0%)	2.1	2 (1–3)
PHE	411	46 (11%)	99 (24%)	122 (30%)	81 (20%)	38 (9%)	17 (4%)	6 (1%)	2 (0%)	2.1	2 (1–3)
SUA	45	8 (18%)	15 (33%)	14 (31%)	4 (9%)	3 (7%)	0 (0%)	1 (2%)	0 (0%)	1.6	1 (1–2)

Abbreviations: CCHFV, Crimean-Congo hemorrhagic fever virus; EBOV, Ebola virus; LASV, Lassa virus; MARV, Marburg virus; PHE, Phebe Airstrip; RVFV, Rift Valley Fever virus; SUA, Suakoko.

## DISCUSSION

In this study of people living in rural, central Liberia, we find evidence of high rates of seropositivity to several disease-causing viral pathogens with most individuals positive for multiple pathogens. This finding has several implications. Foremost, this unexpectedly high seropositivity demonstrates that undetected spillover events are occurring and can be associated with demographic and environmental factors. In addition, seropositivity to these consequential pathogens was detected in people who report no prior serious illness contributing to an evolving understanding of the broad spectrum of disease caused by these infections. Although many spillover events may lead to asymptomatic or minimally symptomatic disease with little clinical consequence, it may also be possible that infection does produce symptomatic disease, sometimes severe, mistakenly diagnosed as malaria, typhoid fever, or other, more common infectious diseases. The 2014 EBOV epidemic is evidence that a single spillover event can lead to devastating epidemics that cross international boundaries and threat global public health.

In this region of Liberia where Lassa virus is endemic, LASV seropositivity was markedly high at 67% and higher than previously described in Sierra Leone (50%), Guinea (60%), and Mali (44%) [11, 17, 18]. Seropositivity increased with age suggesting ongoing exposure throughout life. This can be expected given the endemicity of the *Mastomys natalensis* rats in the region and the potential for human–rodent contact in homes—a finding supported by the trend toward higher LASV seropositivity in those reporting rat holes near their homes. Surprisingly, we found a lack of negative association with LASV seropositivity and cat exposure, which would be expected based on previous work reporting individuals with known cat exposures contribute less to clinical Lassa fever cases [19]. Exposure to LASV outside the home may explain this finding.

High rates of seropositivity were also found for 2 bunyaviruses CCHFV and RVFV, both of which are known to circulate in livestock across sub-Saharan Africa, with occasional spillover into humans [20, 21]. Bong County accounts for 16% of agriculture households in Liberia with livestock (predominantly goats and sheep) and imported approximately 13 000 goats and sheep in 2019 [22]. Prior studies in West Africa revealed significant seropositivity in goats and sheep making them potential amplifying reservoirs in this region of Liberia [23, 24]. Additionally, domestic animals such as dogs and cats have been identified as potential reservoirs for both viruses, which may explain the higher seropositivity among participants reporting cat exposures [25]. In this Liberian cohort, CCHFV seropositivity reached 24%, much higher than historically reported in neighboring countries including Sierra Leone (2%) and Guinea (3%), with some studies prior to 2016 detecting no CCHFV seropositivity at all [11, 21, 25]. Combined with our findings, these results suggest that CCHFV spillover events in West Africa may be increasing. There is weak cross-reactivity between antibodies targeting CCHFV NP and other Nairo viruses [26, 27]. As for RVFV seropositivity in our cohort was 9%, which is in line with the 11% found in Sierra Leone in 2014 [11].

EBOV seropositivity was higher at 13% compared to 5%–9% reported in Sierra Leone and Guinea [28]. The higher than expected seropositivity reported here was likely in part driven by the cumulative incident rate of EBOV disease (>300 per 100 000) observed in Bong County during the 2014–2016 EBOV epidemic [29]. Additionally, environmental encroachment, bushmeat hunting, and occupational exposures, often male-dominated activities, are likely contributing factors. Most importantly, the high seropositivity in the context of only 3 participants reporting prior EBOV disease suggests that most of these infections were either mild or asymptomatic. EBOV seropositivity has also been noted in household contacts of Ebola virus disease survivors who report no prior diagnosis of this disease [30].

MARV seropositivity in the current study was comparable to reports from surrounding countries [11]. This is an unexpected result, as to date no MARV outbreaks have been reported in Liberia, unlike neighboring Guinea and Sierra Leone, suggesting most cases are likely mild or asymptomatic, or, as mentioned previously, severe cases are potentially being misclassified. Of note, there were very few participants with seropositivity to both MARV and EBOV, reflecting either differences in risk factors or potentially infection with 1 filovirus conferring some protection to others; a hypothesis supported by EBOV survivors possessing antibodies with some cross-reactivity with Marburg virus glycoproteins [31].

Rates of seropositivity for flaviviruses and alphaviruses, which are known to cause disease across West Africa, were high, as expected. In Guinea, Nigeria, and Cameroon, seropositivity is common for flavivirus and alphavirus including yellow fever virus (27–43%), Dengue virus (12–45%), and West Nile virus (7–49%) [32–34]. In the cohort presented here, 51% were seropositive for flaviviruses and 35% for alphaviruses; however, we used pan-flavivirus and pan-alphavirus assays, which precludes the ability to determine seropositivity for individual viruses [11]. Our finding of increased pan-alphavirus seropositivity with age suggests ongoing exposure over time. Interestingly, we observed lower flavivirus seropositivity among participants reporting cat exposure, possibly pointing to their role in controlling local reservoirs such as rodents or birds.

A strength of the current study design was the collection of serum from asymptomatic community members, rather than from individuals seeking care for febrile illnesses, as has been common in prior studies. We believe this approach is likely to provide a more accurate estimate of seropositivity in the general population and captures prior infections that may have been asymptomatic or mild. However, our findings are limited to only 2 rural communities in Liberia, limiting generalizability to other regions of Liberia, particularly urban settings.

The MAGPIX assay represents an additional strength. Compared to standard enzyme-linked immunosorbent assays, previously used, the MAGPIX assay is significantly more sensitive and able to detect LASV and EBOV antibodies 10 times less concentrated than enzyme-linked immunosorbent assays [35]. The multiplex nature allows for increased throughput and faster time to answer [36, 37]; also, reduced costs from smaller sample volumes and fewer reagent requirements. All of which are vital for improving surveillance in resource poor environments. However, the MAGPIX assay does have limitations; primarily the lack of standardized methodology for establishing SNR thresholds for defining seropositivity in a multiplexed assay from a cross-sectional study.

In a cross-sectional study of IgG seropositivity from community surveillance, definitive exposure of the population to pathogens is a true unknown. These assays are developed and verified with animal model samples and known human positives to establish well-controlled seropositivity thresholds [38]. However, we find with cross-sectional cohorts, these thresholds must be set more conservatively. Furthermore, establishing clear correlations between SNR thresholds and neutralizing antibodies for these hemorrhagic fever viruses will be crucial for improving interpretation of future studies. Although neutralizing antibodies are often a fraction of the total pathogen specific antibody prevalence in a sample, a better understanding of potential correlation of SNR threshold to viral neutralization will assist in these studies. The difficulty in this is the cost, time, sample requirement, and biosafety constraints needed for neutralization studies on hundreds of samples at the BSL3/4 level.

Another limitation is the focus of the questionnaire on LASV exposure risk factors. The questionnaire was developed to facilitate the aims of the LASV focused ENABLE protocol prior to the design of this exploratory study. Despite this, the study still identified factors associated with seropositivity for most of the pathogens.

In conclusion, we find evidence of widespread exposure to high consequence viral pathogens in this region of West Africa, raising concern that zoonotic spillover events are occurring, but may lead to minimally symptomatic disease or more severe but underdiagnosed illness. Such cases could serve as a nidus for larger outbreaks, epidemics and pandemics. Exploratory studies such as this provide data used to improve diagnostic capacity by identifying which pathogens are circulating, what additional clinical diagnostic testing should be prioritized (ie, polymerase chain reaction testing for circulating pathogens), and what environmental surveillance is needed (ie, local livestock or domesticated animals). At the same time, sero-surveillance studies can help identify populations that can benefit from measures, such as vaccination, which keep transmission of these pathogens from becoming outbreaks or pandemics.

## Supplementary Material

ofag100_Supplementary_Data

## Supplementary Data

Supplementary materials are available at *Open Forum Infectious Diseases* online. Consisting of data provided by the authors to benefit the reader, the posted materials are not copyedited and are the sole responsibility of the authors, so questions or comments should be addressed to the corresponding author.
