# Supplementary material for: Serologic Evidence of Hemorrhagic Fever Virus Spillover in Rural Liberia
Source: Open Forum Infect Dis. 2026 Mar 4;13(3):ofag100. doi: 10.1093/ofid/ofag100 (PMC13012602; doi:10.1093/ofid/ofag100)

**Supplemental Materials**

**Detailed Methodology for MAGPIX Assay**

The following capture antigens were covalently linked to magnetic microspheres: Pan-Flavivirus virus like particle, EBOV nucleoprotein; Pan-alphavirus virus like particle; CCHFV nucleoprotein; LASV nucleoprotein; MARV glycoprotein; RVFV nucleoprotein. Important to note we utilized the EBOV NP over VP40 and GP as it is the more conserved immunodominant protein. Bead samples were vortexed and diluted 1:250 in phosphate-buffered saline with Tween 20 (PBST). Fifty microliters of diluted capture beads were added to wells of a 96-well plate which were placed on a Luminex magnetic plate and incubated for 60 seconds before discarding buffer. Participant samples were then diluted 1:100 in PBST alongside negative and positive controls and added to the appropriate wells in duplicate on the antigen coated 96-well plate. Plates were placed on shaker for 1 hour then washed with 100 µl of PBST three times. Next human anti-IgG-PE was diluted 1:100 in PBST and 50 µL was added to each well. Plates were placed on shaker for 1 hour and then liquid was discarded. Wells were then washed three times with 100 µl of PBST.

**Figure S1:** Mean fluorescence intensity (MFI) signal to noise ratio (SNR) distribution plot for all samples evaluated for CCHF, EBOV, LASV, MARV, Pan-alphavirus, Pan-flavivirus and RVFV antibodies. Red line represents a threshold using the SNR threshold of 3 highlighted in previous work. Dashed line is the SNR of 20 utilized in this study and the solid black line represents the SNR of 40 associated with LASV-specific neutralizing antibodies.


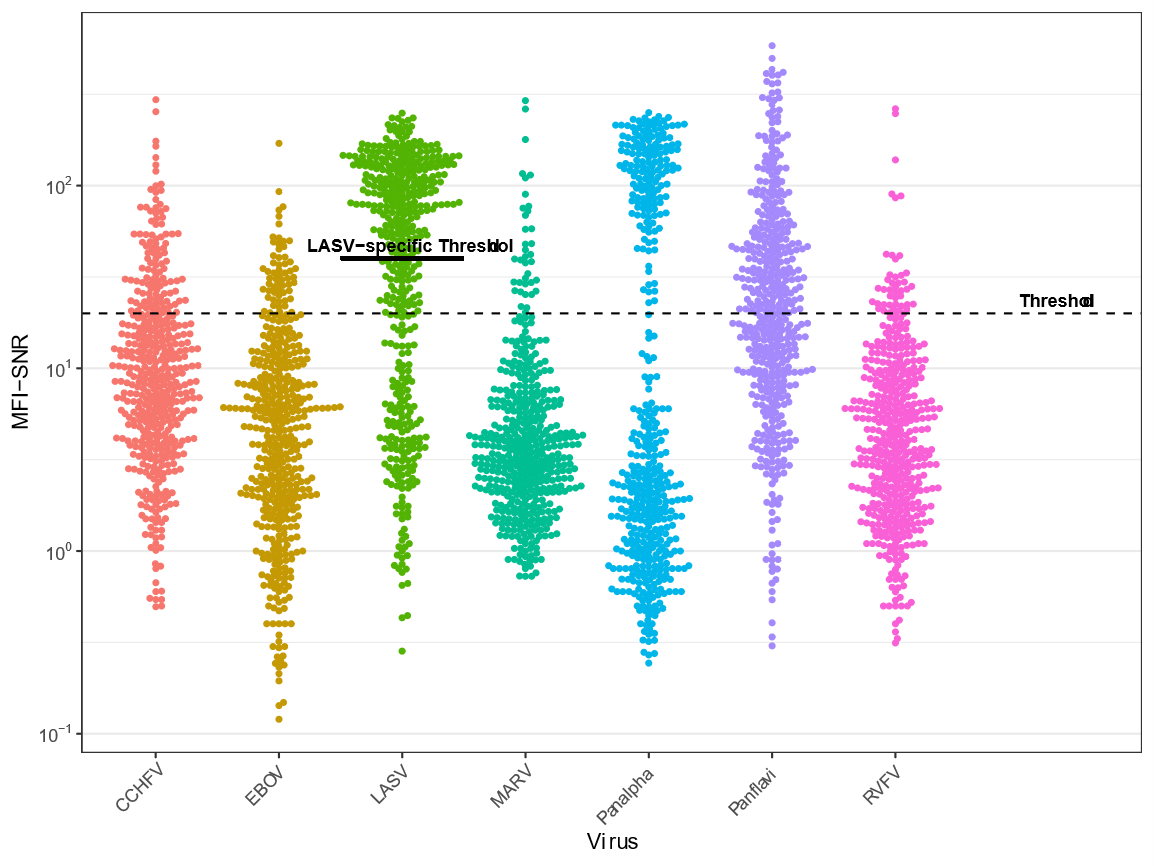


**Figure S2:** Forest plots for baseline associations with virus seropositivity. Demographic and household features were analyzed for each of the seven viral seropositivity’s tested: Lassa virus (S2.1), pan-alphavirus (S2.2), Rift Valley fever virus (S2.3), pan-flavivirus (S2.4), Crimean-Congo hemorrhagic fever virus (S2.5), Marburg virus (S2.6) and Ebolavirus (S2.7). For each factor, seropositivity prevalence differences were estimated between index and referent with a corresponding 95% confidence interval. Age across multiple ranges was compared to children 2-5 years of age (reference range).

Figure S2.1: Forest plot for baseline associations with Lassa virus seropositivity.

**
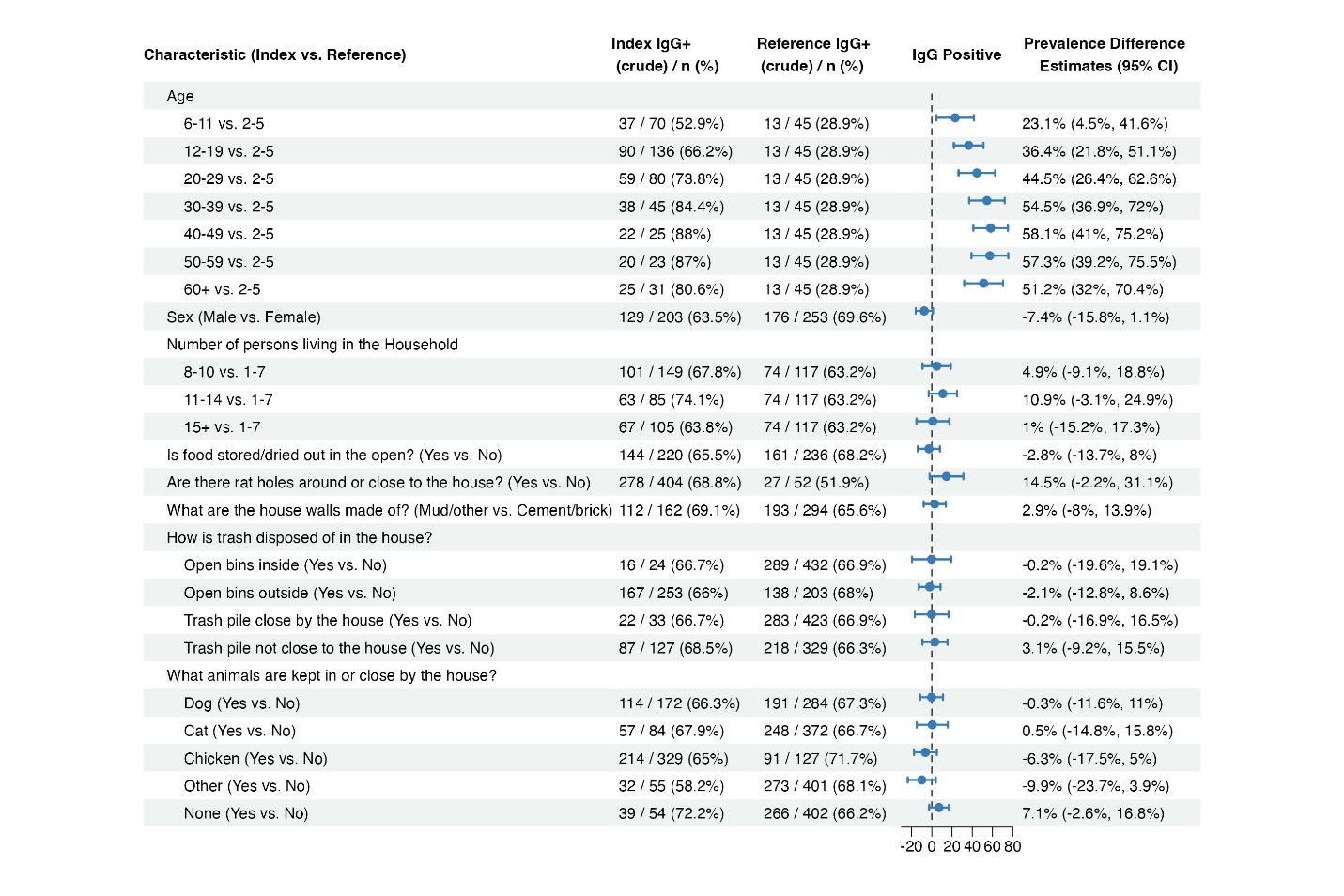
**

Figure S2.2: Forest plot for baseline associations with pan-alphavirus seropositivity.

**
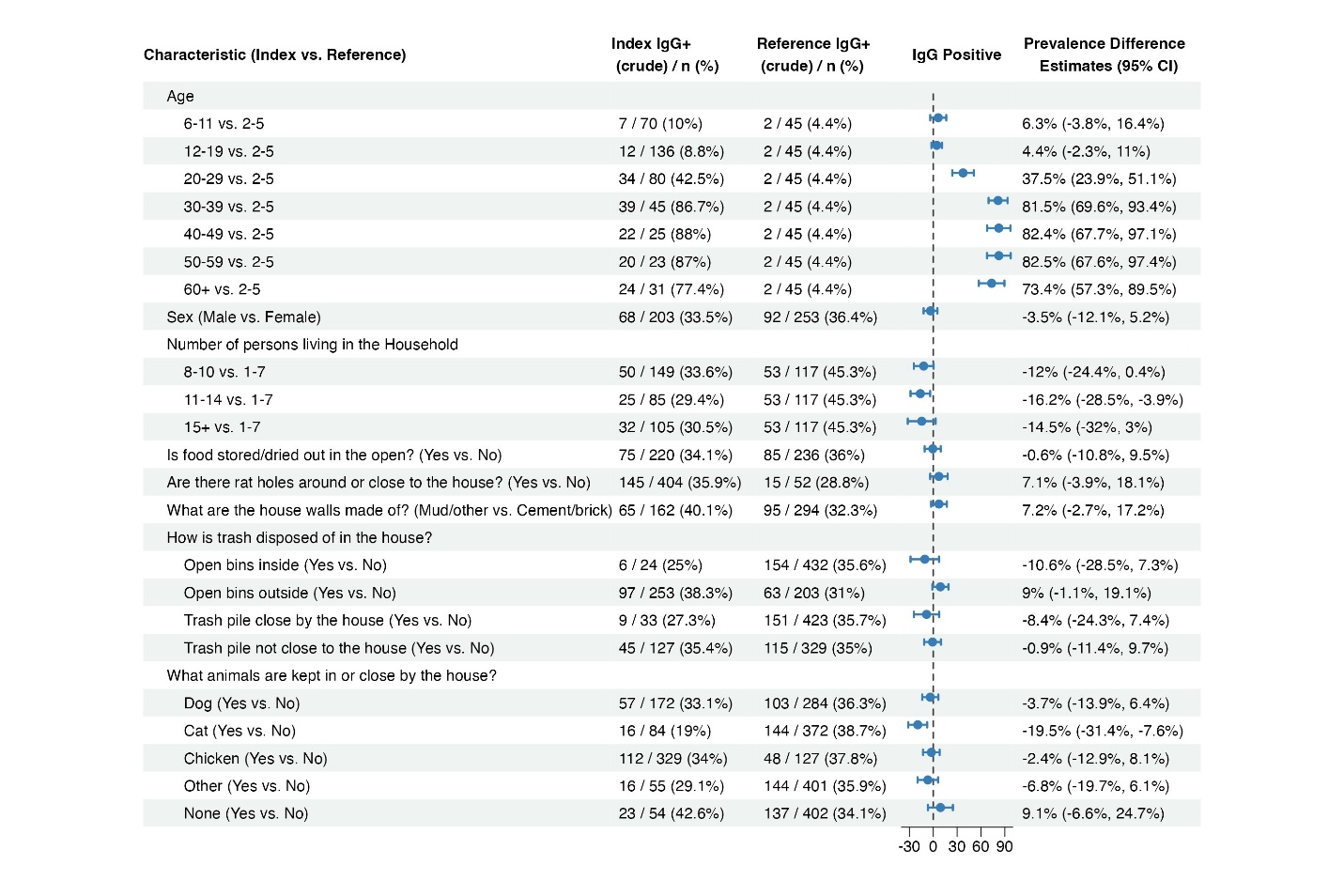
**

Figure S2.3: Forest plot for baseline associations with Rift Valley fever virus seropositivity.

**
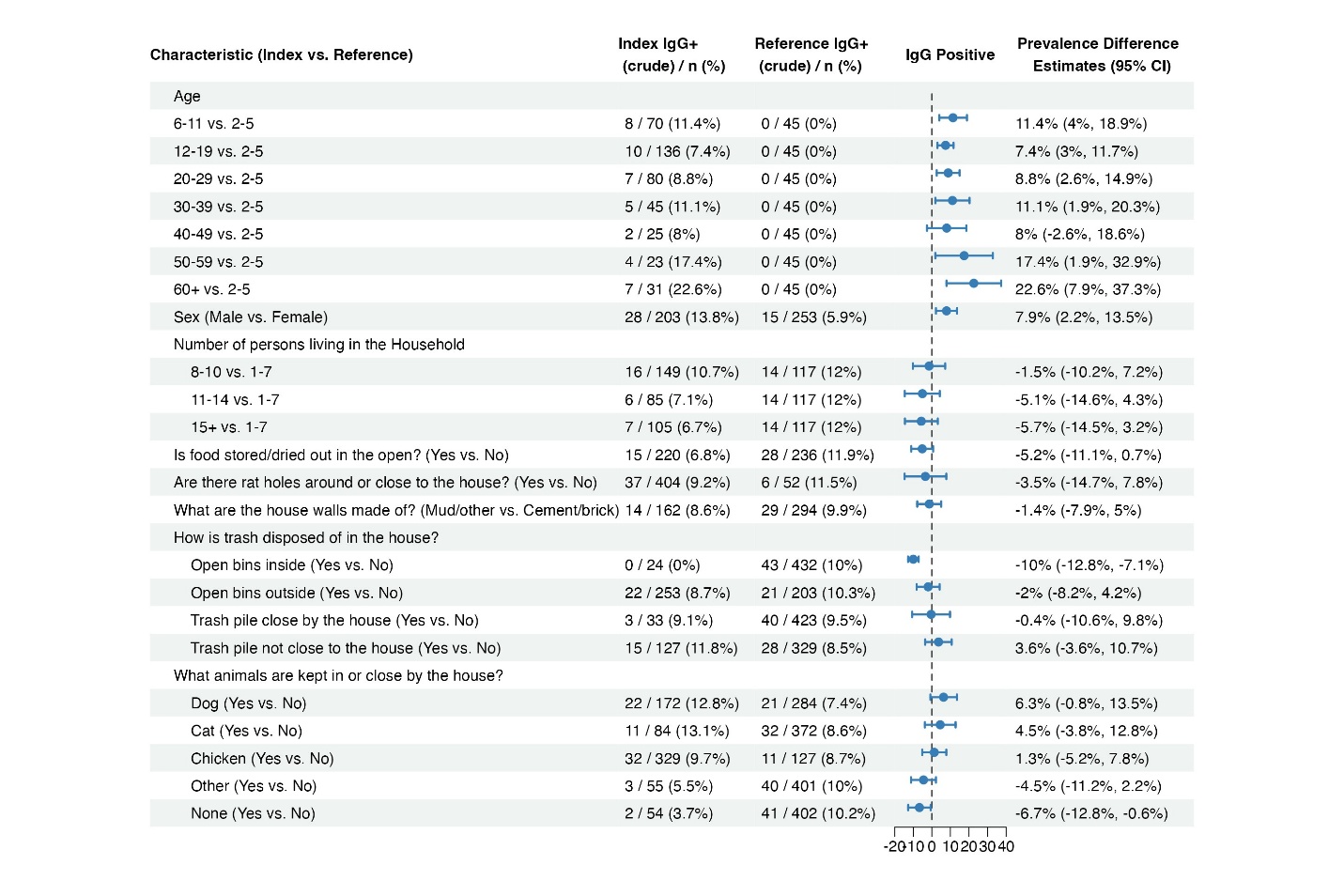
**

Figure S2.4: Forest plot for baseline associations with pan-flavivirus seropositivity.

**
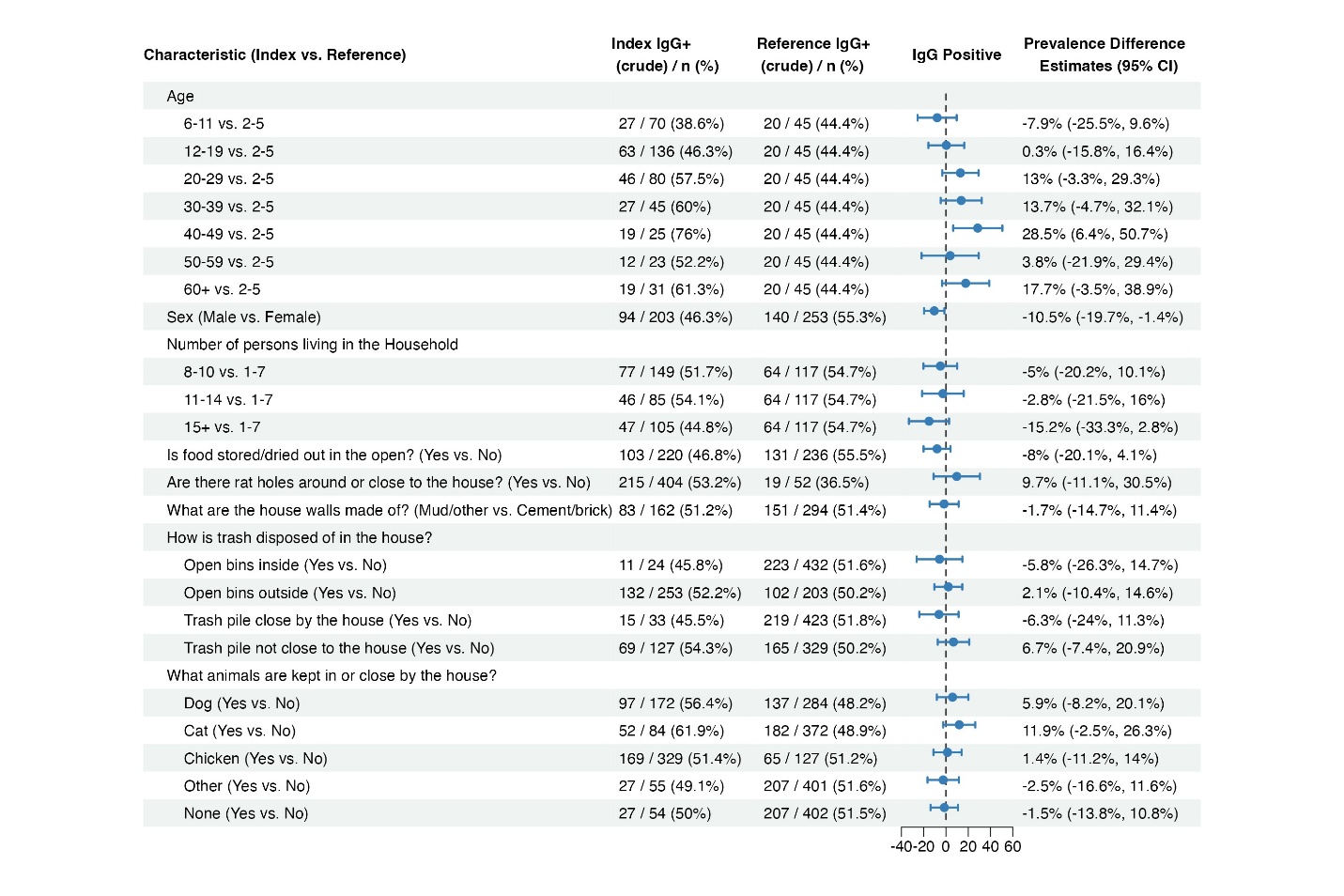
**

Figure S2.5: Forest plot for baseline associations with Crimean-Congo hemorrhagic fever virus seropositivity.

**
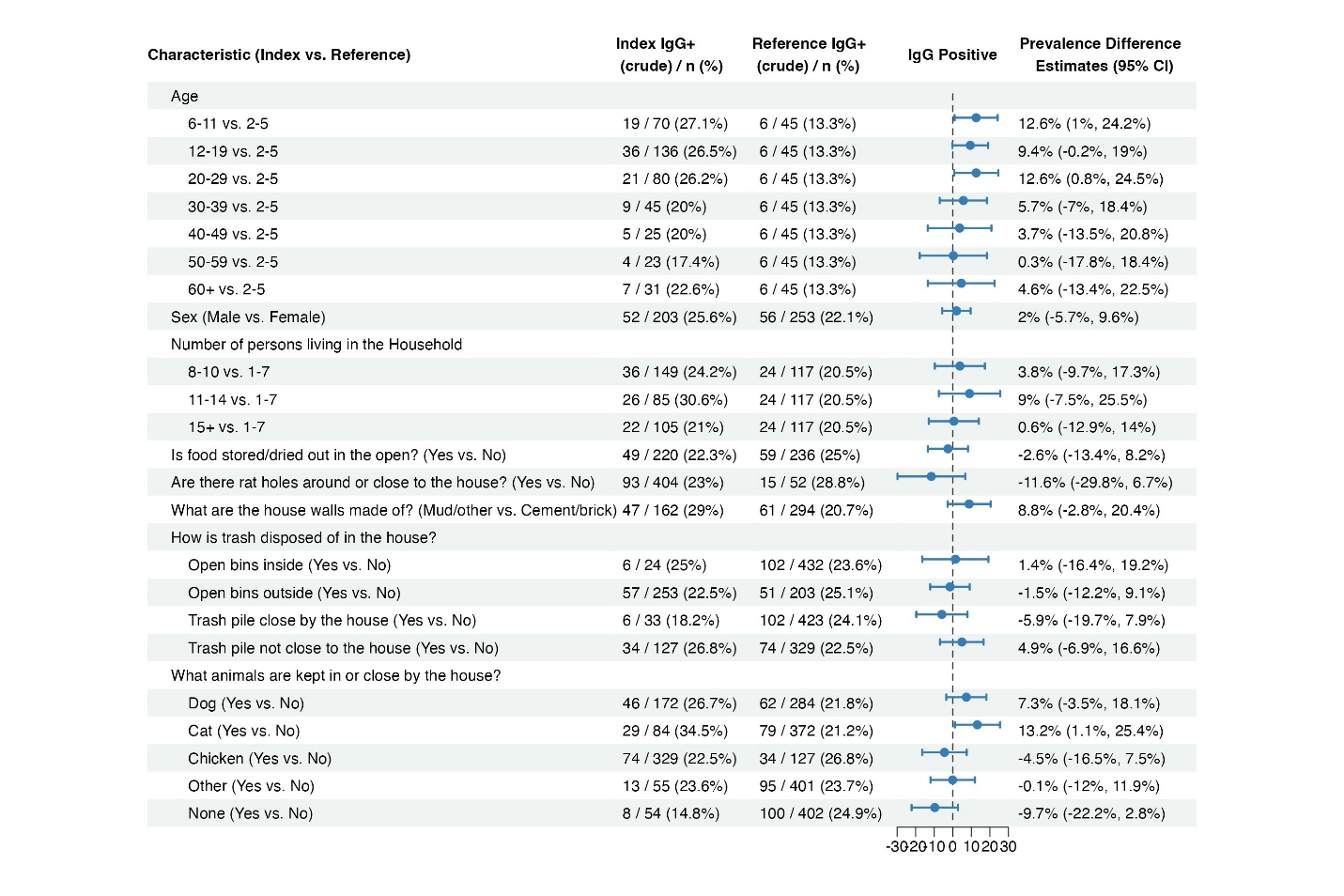
**

Figure S2.6: Forest plot for baseline associations with Marburg virus seropositivity.

**
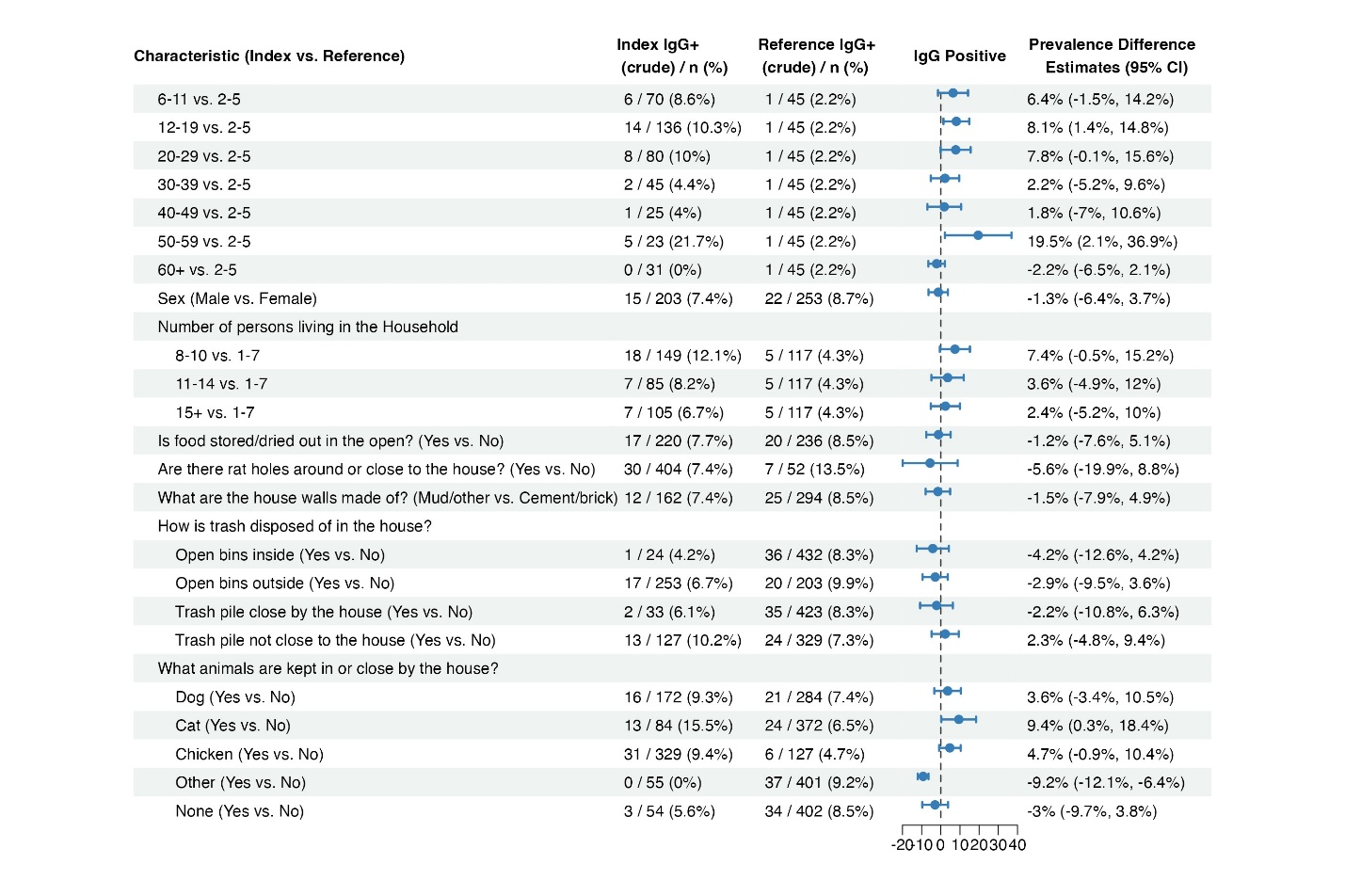
**

Figure S2.7: Forest plot for baseline associations with Ebolavirus seropositivity.

**
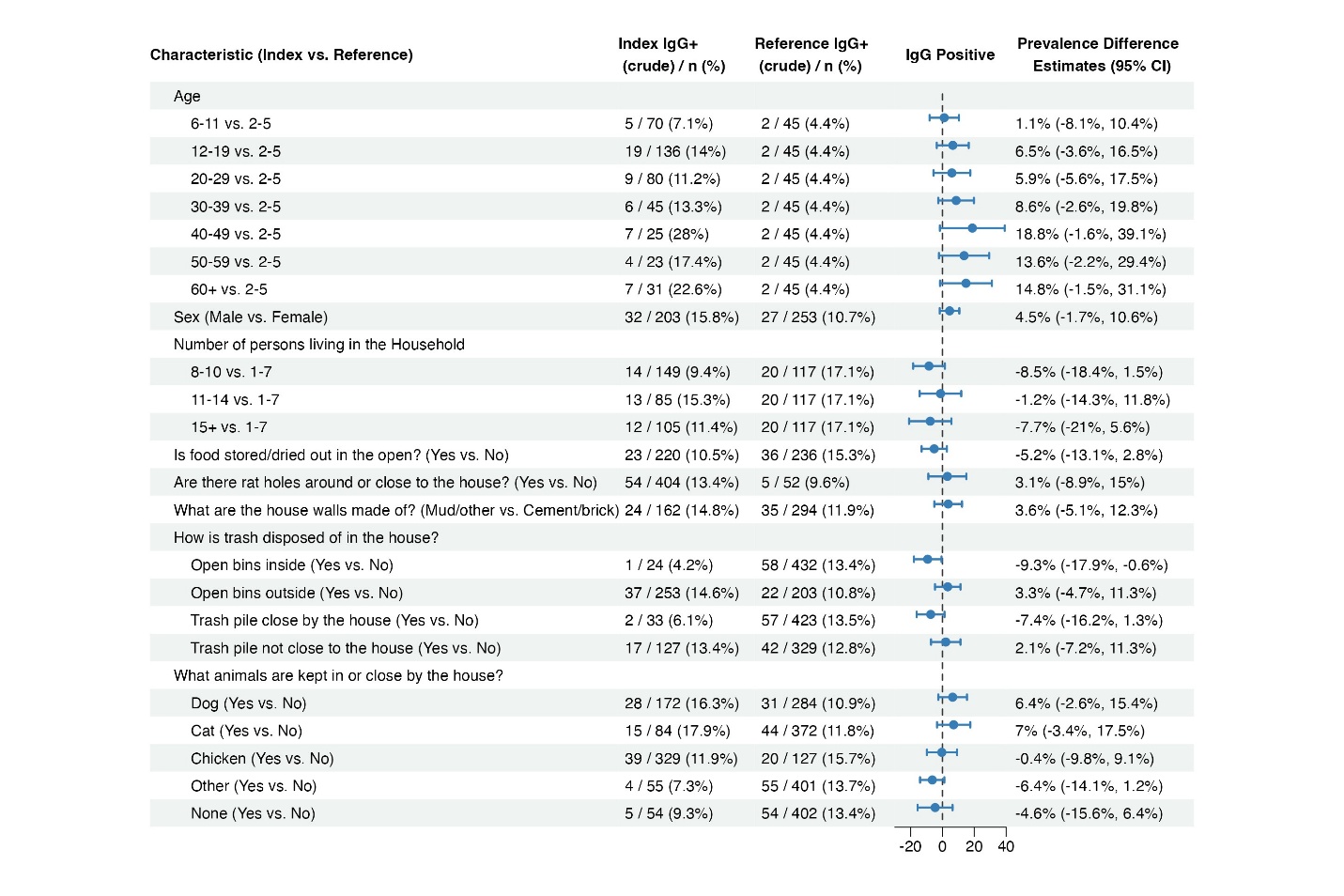
**

**Figure S3**: Spearman rank-based correlation heatmap visualizes associations among raw MFI-SNR values. Values range from -1 (negative correlation), 0 (no correlation) and 1 (positive correlation).


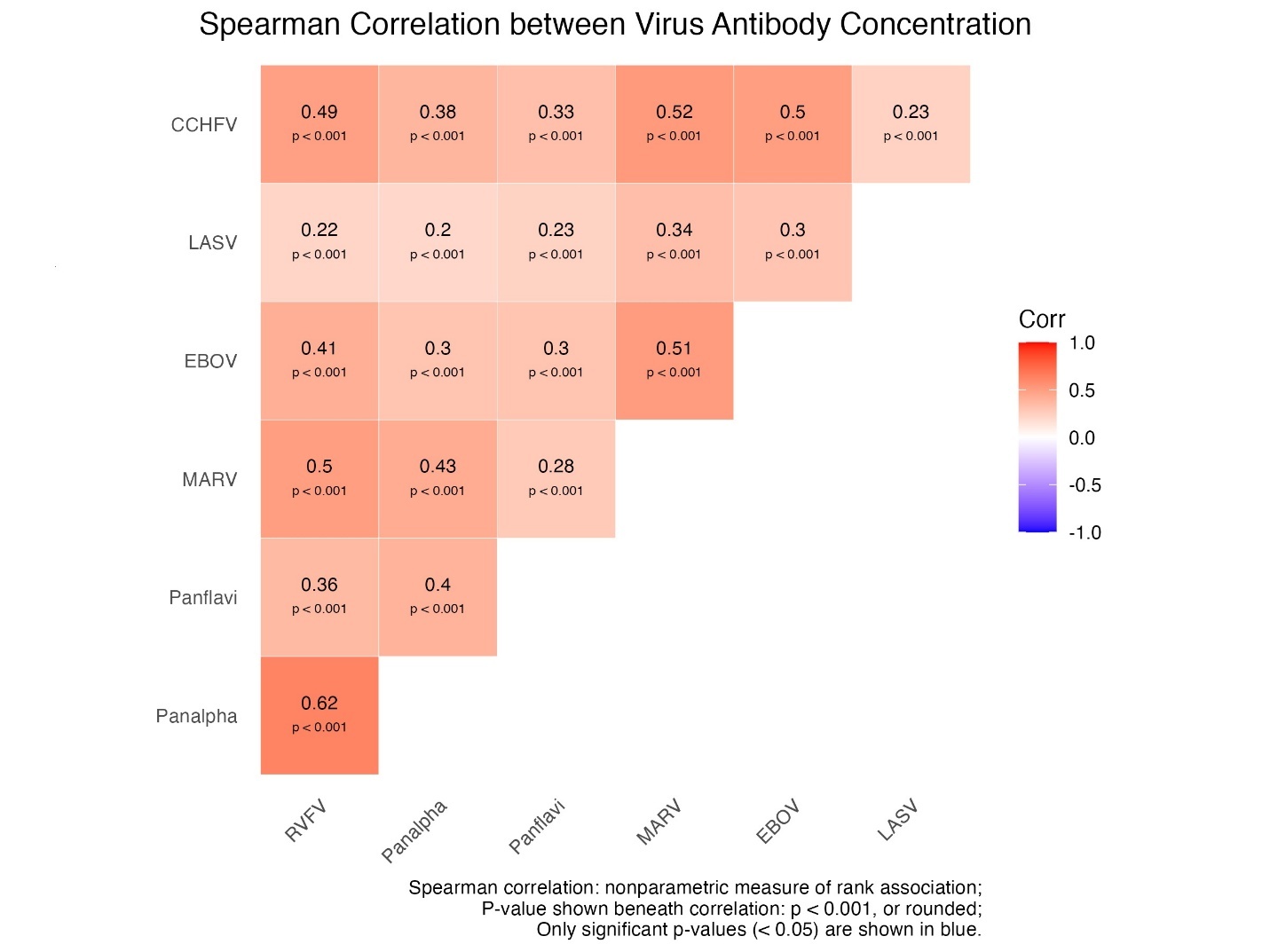

Supplement: ofag100_Supplementary_Data [file ofag100_supplementary_data.docx]
